# Supplementary material for: Prevalence and clinical characteristics of increased pancreatic enzymes in patients with severe fever with thrombocytopenia syndrome
Source: PLoS Negl Trop Dis. 2023 Nov 9;17(11):e0011758. doi: 10.1371/journal.pntd.0011758 (PMC10662747; doi:10.1371/journal.pntd.0011758)
Supplement: S1 Checklist — (DOC) [file pntd.0011758.s001.doc]

**S1 Checklist. STROBE checklist.**

|  | Item No | Recommendation |
| --- | --- | --- |
| **Title and abstract** | 1 | (*a*) A retrospective observational study |
|  |  | (*b*) The aim of this study was to explore the prevalence, clinical characteristics of elevated pancreatic enzymes (amylase and lipase) and its association with AP in patients with SFTS. We found that the increased pancreatic enzymes are very common in patients with STFS, but they are not always associated with AP. AP is related to high mortality rate of patients with SFTS, accounting for the majority of deaths of people with elevated pancreatic enzymes. |
| Introduction | | |
| Background/rationale | 2 | The pancreatic injury has recently been reported in patients with severe fever with thrombocytopenia syndrome (SFTS). However, its significance has not been elucidated clearly. |
| Objectives | 3 | The aim of this study was to explore the prevalence, clinical characteristics of elevated pancreatic enzymes (amylase and lipase) and its association with AP in patients with SFTS. |
| Methods | | |
| Study design | 4 | A retrospective observational study |
| Setting | 5 | Data of demographics, comorbid conditions, clinical symptoms, laboratory parameters and survival time of patients with SFTS were collected. Patients were assigned into the non-AP and AP groups according to the diagnostic criteria of AP. Patients in the non-AP group were divided into the normal (<upper limit of normal [ULN]), elevated pancreatic enzymes (EPE) (1-3×ULN) and high pancreatic enzymes (HPE) (>3×ULN) groups according to the serum amylase and lipase levels, and then their clinical data were compared. |
| Participants | 6 | A total of 284 consecutive patients with SFTS admitted to the Department of Infectious Disease, Zhongnan Hospital of Wuhan University between August 2016 and June 2023 were enrolled in a retrospective cohort. |
| Variables | 7 | The criteria of diagnosing SFTS were as follows: febrile patients (temperatures of 37.3 ˚C or more for over 24 hours) and decreased platelet; laboratory-confirmed SFTSV infection by detection of viral RNA in serum via reverse transcriptase polymerase chain reaction. AP diagnostic criteria were referred to the revision of the Atlanta criteria. Diagnosis of AP met at least two of the following three features: (1) serum amylase and/or lipase levels≥3×ULN; (2) abdominal pain (acute onset of persistent, severe, epigastric pain often radiating to the back); and (3) characteristic findings of AP on imaging examinations. |
| Data sources/ measurement | 8* | The medical records of patients with SFTS were reviewed, demographic details, comorbid conditions, symptoms, signs, radiological findings and laboratory tests data including white blood cell (WBC) count and percentage, neutrophils count and percentage, lymphocyte count and percentage, platelet count, hemoglobin, alanine aminotransferase (ALT), aspartate aminotransferase (AST), total bilirubin (TBIL), albumin, globulin, alkaline phosphatase (ALP), gamma glutamyl transpeptidase (GGT), lactate dehydrogenase (LDH), amylase, lipase, blood urea nitrogen (BUN), creatinine, cystatin C, sodium, potassium, calcium, creatinine kinase (CK), creatinine kinase myocardial b fraction (CK-MB), troponin I, brain natriuretic peptide (BNP), prothrombin time (PT), international normalized ratio (INR), prothrombin activity (PTA), activated partial thromboplastin time (APTT), thrombin time (TT), fibrinogen, D-dimer, C-reactive protein (CRP), procalcitonin, interleukin-6 (IL-6), erythrocyte sedimentation rate (ESR), SFTSV viral load, occult blood test (OBT) and survival time were collected. |
| Bias | 9 | This was a retrospective single-center study, the observations made here could not be extrapolated to other centers. |
| Study size | 10 | A total of 284 patients diagnosed with SFTS were retrospectively enrolled, including 248 patients in the non-AP group and 36 patients in the AP group. Patients in the non-AP group were composed of 48, 116 and 84 patients in the normal, EPE and HPE groups, respectively. |
| Quantitative variables | 11 | The medical records of patients with SFTS were reviewed, demographic details, comorbid conditions, symptoms, signs, radiological findings and laboratory tests data were collected. |
| Statistical methods | 12 | Categorical variables were shown as numbers (percentages) and were compared by the Chi-square test or Fisher’s exact test. Continuous variables were shown as the means ± standard deviations for data with a normal distribution or medians with interquartile ranges (P25-P75) for data with a non-normal distribution, which were compared by the Student’s t test or Mann–Whitney U test, respectively. The cumulative survival rates of patients were evaluated using the Kaplan-Meier method and were compared by the Log-rank test. Analysis of receiver operating characteristic (ROC) curves was used to calculate the area under the curve (AUC), and Youden index was used to identify the suggested cutoff value for diagnosing AP in SFTS. All data were analyzed with IBM SPSS statistical analysis software (version 26.0, Chicago, USA), and P < 0.05 (two-sided) was considered statistically significant. |
| Results | | |
| Participants | 13* | 393 patients hospitalized for “SFTS” between August 2016 and June 2023, 109 patients were excluded for one or more the following reasons: 56 patients did not satisfy the criteria of diagnosing SFTS, 24 patients lose the follow-up, 12 patients had incomplete data, 9 patients had severe preterminal comorbidities, 8 patients had biliary stones, alcoholism or hyperlipidemia, a total of 284 patients were enrolled into this study. |
| Descriptive data | 14* | A total of 284 patients diagnosed with SFTS were retrospectively enrolled, including 248 patients in the non-AP group and 36 patients in the AP group. Patients in the non-AP group were composed of 48, 116 and 84 patients in the normal, EPE and HPE groups, respectively. |
| Outcome data | 15* | The cumulative survival rates of patients in the normal, EPE, HPE and AP groups were 95.8%, 89.7% and 83.3%, 41.7%,respectively. |
| Main results | 16 | A total of 284 patients diagnosed with SFTS were retrospectively enrolled, including 248 patients in the non-AP group and 36 patients in the AP group. Patients in the non-AP group were composed of 48, 116 and 84 patients in the normal, EPE and HPE groups, respectively. Compared with patients in the normal and EPE groups, patients in the HPE group had higher serum levels of laboratory parameters referring to liver, kidney, heart and coagulation system injury, as well as higher viral load. The cumulative survival rate of patients in the HPE group was significantly lower than that of patients in the normal group. In addition, patients in the AP group also had higher serum levels of laboratory variables reflecting liver, heart, coagulation dysfunction and viral load than patients in the HPE group. The cumulative survival rate of patients in the AP group was significantly lower than that of patients in the HPE group. |
| Other analyses | 17 | Serum amylase and lipase were used to diagnose AP in patients with SFTS. The AUC of amylase was 0.965 (95% CI 0.938–0.992). With a cutoff value of 260 U/L, the sensitivity and specificity of amylase were 94.4% and 91.5%, respectively. The positive predictive value was 61.9%, and the negative predictive value was 99.1%. The AUC of lipase was 0.938 (95% CI 0.896-0.981). With a cutoff value of 406 U/L, the sensitivity and specificity of lipase were 83.3% and 92.3%, respectively. The positive predictive value was 61.2%, and the negative predictive value was 97.4%. |
| **Discussion** |  |  |
| Key results | 18 | In this present study, we revealed that the incidence of elevated pancreatic enzymes without AP in SFTS was up to 70.4%, it demonstrated that the elevation of pancreatic enzymes without AP was very common in patients with SFTS. Among patients without AP, we reported that patients with pancreatic enzymes >3×ULN had significantly higher serum levels of laboratory parameters referring to liver, kidney, heart, coagulation system injury and viral load than patients with pancreatic enzymes <3×ULN. Furthermore, we also demonstrated that patients with AP had significantly more presence of consciousness disorder, higher serum levels of laboratory variables reflecting liver, heart, coagulation dysfunction and viral load than patients with pancreatic enzymes >3×ULN without AP. Among SFTS patients without AP, we showed that the cumulative survival rate of patients with pancreatic enzymes >3×ULN was significantly lower than that of patients without elevated pancreatic enzymes. Additionally, we found that the serum levels of amylase and/or lipase of patients diagnosed with AP were all higher than 3×ULN. The cumulative survival rate of patients with AP was significantly lower than that of patients with pancreatic enzymes >3×ULN without AP, and AP was an independent predictor of mortality for patients with SFTS. It suggested that AP might account for the majority of deaths of SFTS people with elevated pancreatic enzymes. |
| Limitations | 19 | The main limitations of our study contain the retrospective study design. In addition, the sample size of this study was relatively small. Therefore, the subsequent analysis of risk factors for AP development in SFTS was not conducted. Finally, because this was a single-center study, the observations made here could not be extrapolated to other centers. |
| Interpretation | 20 | In summary, clinicians should be aware that the elevated pancreatic enzymes are very common in SFTS, and may not always represent a true AP. Imaging examinations could be necessary for clinicians to confirm the diagnosis of AP when SFTS patient with elevated pancreatic enzymes. Though AP may account for the majority of mortality of patients with elevated pancreatic enzymes, patients with pancreatic enzymes >3×ULN except for AP also had a high in-hospital mortality rate. |
| Generalisability | 21 | The elevated pancreatic enzymes are very common in SFTS, and may not always represent a true AP. Imaging examinations could be necessary for clinicians to confirming the diagnosis of AP when SFTS patient with elevated pancreatic enzymes. |
| **Other information** |  |  |
| Funding | 22 | This work was supported by grants from Key Research and Development Program of Hubei Province, China (2020BCB025). |

*Give information separately for cases and controls in case-control studies and, if applicable, for exposed and unexposed groups in cohort and cross-sectional studies.

**Note:** An Explanation and Elaboration article discusses each checklist item and gives methodological background and published examples of transparent reporting. The STROBE checklist is best used in conjunction with this article (freely available on the Web sites of PLoS Medicine at http://www.plosmedicine.org/, Annals of Internal Medicine at http://www.annals.org/, and Epidemiology at http://www.epidem.com/). Information on the STROBE Initiative is available at www.strobe-statement.org.
