# Supplementary material for: Prevalence and clinical characteristics of increased pancreatic enzymes in patients with severe fever with thrombocytopenia syndrome
Source: PLoS Negl Trop Dis. 2023 Nov 9;17(11):e0011758. doi: 10.1371/journal.pntd.0011758 (PMC10662747; doi:10.1371/journal.pntd.0011758)
Supplement: S1 Table — (DOCX) [file pntd.0011758.s002.docx]

**S1 Table. Comparison of demographics, comorbid conditions and clinical symptoms of SFTS patients in the survivor and non-survivor groups.**

|  | Survivor (n=235) | Non-survivor (n=49) | *P* value |
| --- | --- | --- | --- |
| Male, n (%) | 123(52.3) | 20(40.8) | 0.142 |
| Age (years) | 65±8 | 66±8 | 0.084 |
| Diabetes mellitus, n (%) | 15(6.4) | 6(12.2) | 0.154 |
| Hypertension, n (%) | 55(23.4) | 14(28.6) | 0.443 |
| Days from onset to admission | 6(5-8) | 7(5-9) | 0.447 |
| Clinical manifestations, n (%) |  |  |  |
| Fever >38 ℃ | 57(24.3) | 20(40.8) | 0.018 |
| Headache | 41(18.7) | 11(22.4) | 0.410 |
| Dizziness | 77(32.8) | 17(34.7) | 0.794 |
| Cough | 54(23.0) | 13(26.5) | 0.594 |
| Sputum | 41(17.4) | 9(18.4) | 0.878 |
| Chest distress | 44(18.7) | 11(22.4) | 0.548 |
| Anorexia | 160(68.1) | 37(75.5) | 0.305 |
| Nausea | 154(65.5) | 34(69.4) | 0.604 |
| Vomiting | 74(31.5) | 14(28.6) | 0.688 |
| Abdominal pain | 53(22.6) | 26(53.1) | <0.001 |
| Diarrhea | 54(23.0) | 13(26.5) | 0.594 |
| Petechia | 15(6.4) | 12(24.5) | <0.001 |
| Consciousness disorder | 21(8.9) | 22(44.9) | <0.001 |
| Hepatosplenomegaly | 14(6.0) | 11(22.4) | <0.001 |
