# Supplementary material for: Prevalence and clinical characteristics of increased pancreatic enzymes in patients with severe fever with thrombocytopenia syndrome
Source: PLoS Negl Trop Dis. 2023 Nov 9;17(11):e0011758. doi: 10.1371/journal.pntd.0011758 (PMC10662747; doi:10.1371/journal.pntd.0011758)
Supplement: S2 Table — (DOCX) [file pntd.0011758.s003.docx]

**S2 Table. Comparison of laboratory parameters of SFTS patients in the survivor and non-survivor groups.**

| Variables | Normal range | Survivor (n=235) | Non-survivor (n=49) | *P* value |
| --- | --- | --- | --- | --- |
| WBC (10^9^/L) | 3.5-9.5 | 3.7(2.1-6.6) | 3.9(2.7-6.4) | 0.828 |
| Neutrophils (10^9^ /L) | 1.8-6.3 | 2.2(1.1-4.8) | 2.9(1.2-5.0) | 0.650 |
| Neutrophils (%) | 40.0-75.0 | 68.6(52.1-81.9) | 69.9(58.7-82.5) | 0.623 |
| Lymphocyte (10^9^/L) | 1.1-3.2 | 0.7(0.5-1.2) | 0.7(0.4-1.4) | 0.639 |
| Lymphocyte (%) | 20.0-50.0 | 22.0(11.7-33.7) | 21.9(13.9-28.9) | 0.588 |
| Platelet (10^9^ /L) | 125-350 | 42(30-62) | 33(21-57) | 0.003 |
| Hemoglobin (g/L) | 130-175 | 124±20 | 119±21 | 0.090 |
| ALT (U/L) | 9-50 | 63(45-108) | 135(73-246) | <0.001 |
| AST (U/L) | 15-40 | 144(69-290) | 582(272-1083) | <0.001 |
| TBIL(μmol/L) | 5-21 | 11.8(8.7-17.2) | 13.2(9.6-21.3) | 0.128 |
| Albumin (g/L) | 40-55 | 28.7±4.5 | 27.0±3.2 | <0.001 |
| Globulin (g/L) | 20-40 | 26.9±4.6 | 27.3±6.2 | 0.549 |
| ALP (U/L) | 30-120 | 71(55-93) | 107(73-193) | <0.001 |
| GGT (U/L) | 8-57 | 34(21-82) | 56(27-217) | <0.001 |
| LDH (U/L) | 125-243 | 576(338-861) | 1000(823-2066) | <0.001 |
| Amylase (U/L) | 0-90 | 142(94-204) | 241(144-332) | <0.001 |
| Lipase (U/L) | 0-70 | 149(82-270) | 315(171-536) | <0.001 |
| BUN (mmol/L) | 2.8-7.6 | 5.2(3.7-6.7) | 7.2(5.2-12.4) | <0.001 |
| Creatinine (μmol/L) | 64-104 | 71(61-89) | 172(68-282) | <0.001 |
| Cystatin C (mg/L) | 0-1.2 | 1.99(1.41-2.87) | 2.23(1.46-3.22) | 0.289 |
| Sodium (mmol/L) | 137-147 | 135.1±5.3 | 135.5±8.7 | 0.690 |
| Potassium (mmol/L) | 3.5-5.3 | 3.5±0.7 | 4.1±1.0 | <0.001 |
| Calcium (mmol/L) | 2.11-2.52 | 1.96±0.17 | 1.76±0.20 | 0.550 |
| CK (U/L) | 0-171 | 255(96-790) | 986(463-1961) | <0.001 |
| CK-MB (U/L) | 0-25 | 25(14-41) | 77(39-134) | <0.001 |
| Troponin I (pg/mL) | 0-26.2 | 80.0(35.2-189.7) | 231(102-412) | <0.001 |
| BNP (pg/mL) | 0-100 | 59(22-155) | 125(60-348) | 0.001 |
| PT (s) | 9.4-12.5 | 11.3(10.6-12.0) | 11.8(11.2-12.9) | 0.001 |
| INR | 0.85-1.15 | 1.03(0.97-1.10) | 1.08(1.02-1.19) | 0.001 |
| PTA (%) | 80-130 | 99(88-111) | 94(80-104) | 0.022 |
| APTT(s) | 25.1-36.5 | 39.4(33.7-44.9) | 53.7(43.8-63.2) | <0.001 |
| TT(s) | 10.3-16.6 | 17.3(15.9-19.6) | 22.3(18.3-27.3) | <0.001 |
| Fibrinogen(mg/dL) | 238-498 | 256(204-301) | 205(147-245) | <0.001 |
| D-dimer (ng/mL) | 0-500 | 913(412-2004) | 1922(866-3471) | <0.001 |
| CRP (mg/L) | 0-10.0 | 6.8(3.1-13.3) | 19.3(9.5-29.7) | <0.001 |
| Procalcitonin (ng/mL) | 0-0.05 | 0.16(0.06-0.49) | 0.84(0.25-1.93) | <0.001 |
| IL-6 (pg/mL) | 0-7 | 34.6(21.0-59.3) | 55.6(40.9-154.1) | <0.001 |
| ESR (mm/h) | 0-20 | 8(5-14) | 14(7-21) | <0.001 |
| Urine RBC counts | 0-13.1 | 16.1(7.6-39.1) | 79.1(26.9-311.6) | <0.001 |
| Viral load (log_10_ copies/mL) |  | 3.8(3.3-4.3) | 5.4(4.3-6.1) | <0.001 |
| OBT positivity, n (%) |  | 45(19.1) | 15(30.6) | 0.074 |
