# Supplementary material for: Prevalence and clinical characteristics of increased pancreatic enzymes in patients with severe fever with thrombocytopenia syndrome
Source: PLoS Negl Trop Dis. 2023 Nov 9;17(11):e0011758. doi: 10.1371/journal.pntd.0011758 (PMC10662747; doi:10.1371/journal.pntd.0011758)
Supplement: S3 Table — (DOCX) [file pntd.0011758.s004.docx]

**S3 Table. Univariable and multivariable logistic regression analyses of in-hospital mortality of patients with SFTS.**

|  | Univariable analysis | | Multivariable analysis | |
| --- | --- | --- | --- | --- |
|  | OR (95% CI) | *P* value | OR (95% CI) | *P* value |
| Male | 0.628(0.336-1.173) | 0.144 |  |  |
| Age(years) | 1.033(0.996-1.071) | 0.085 |  |  |
| Diabetes mellitus | 2.047(0.752-5.572) | 0.161 |  |  |
| Hypertension | 1.309(0.657-2.609) | 0.444 |  |  |
| Days from onset to admission | 1.019(0.922-1.127) | 0.707 |  |  |
| Fever >38 ℃ | 1.978(1.035-3.779) | 0.039 |  |  |
| Abdominal pain | 2.183(1.201-3.949) | 0.021 |  |  |
| Petechia | 1.757(1.063-2.965) | 0.045 |  |  |
| Encephalopathy | 8.303(4.043-22.051) | <0.001 | 8.257(2.238-19.466) | 0.002 |
| Hepatosplenomegaly | 2.570(1.931-5.814) | 0.007 |  |  |
| AP | 3.782(1.415-19.043) | <0.001 | 4.183(1.085-16.121) | 0.038 |
| WBC (10^9^/L) | 0.960(0.865-1.065) | 0.539 |  |  |
| Neutrophils (10^9^ /L) | 0.960(0.865-1.065) | 0.443 |  |  |
| Neutrophils (%) | 1.001(0.993-1.006) | 0.905 |  |  |
| Lymphocyte (10^9^/L) | 1.059(0.694-1.616) | 0.790 |  |  |
| Lymphocyte (%) | 0.989(0.967-1.012) | 0.358 |  |  |
| Platelet (10^9^ /L) | 0.977(0.962-0.993) | 0.004 |  |  |
| Hemoglobin (g/L) | 0.988(0.973-1.002) | 0.094 |  |  |
| ALT (U/L) | 1.003(1.000-1.006) | 0.021 |  |  |
| AST (U/L) | 1.002(1.001-1.003) | <0.001 |  |  |
| TBIL (μmol/L) | 1.010(0.997-1.0324) | 0.132 |  |  |
| Albumin (g/L) | 0.861(0.797-0.931) | <0.001 |  |  |
| Globulin (g/L) | 1.019(0.957-1.086) | 0.548 |  |  |
| ALP (U/L) | 1.006(1.003-1.010) | <0.001 |  |  |
| GGT (U/L) | 1.003(1.001-1.005) | 0.006 |  |  |
| LDH (U/L) | 1.002(1.001-1.003) | <0.001 | 1.002(1.001-1.003) | 0.027 |
| BUN (mmol/L) | 1.148(1.018-1.219) | <0.001 |  |  |
| Creatinine (μmol/L) | 1.015(1.010-1.020) | <0.001 | 1.012(1.002-1.022) | 0.023 |
| Sodium (mmol/L) | 0.989(0.935-1.045) | 0.688 |  |  |
| Potassium (mmol/L) | 3.337(1.953-5.701) | <0.001 |  |  |
| Calcium (mmol/L) | 0.003(0.001-0.027) | <0.001 |  |  |
| CK (U/L) | 1.002(1.001-1.003) | 0.004 |  |  |
| CK-MB (U/L) | 1.012(1.007-1.017) | <0.001 |  |  |
| Troponin I (pg/mL) | 1.002(1.001-1.004) | 0.029 |  |  |
| BNP (pg/mL) | 1.003(0.998-1.005) | 0.137 |  |  |
| PT (s) | 1.010(0.965-1.057) | 0.663 |  |  |
| INR | 1.003(0.990-1.017) | 0.632 |  |  |
| PTA (%) | 0.978(0.961-0.996) | 0.018 |  |  |
| APTT(s) | 1.078(1.049-1.109) | <0.001 |  |  |
| TT(s) | 1.097(1.042-1.156) | <0.001 |  |  |
| Fibrinogen(mg/dL) | 0.988(0.983-0.993) | <0.001 |  |  |
| D-dimer (ng/mL) | 1.001(0.997-1.002) | 0.104 |  |  |
| CRP (mg/L) | 1.027(1.012-1.042) | <0.001 | 1.061(1.031-1.093) | <0.001 |
| Procalcitonin (ng/mL) | 1.039(0.998-1.080) | 0.053 |  |  |
| IL-6 (pg/mL) | 1.004(1.002-1.009) | 0.013 | 1.002(1.003-1.005) | 0.011 |
| ESR (mm/h) | 1.024(1.003-1.046) | 0.025 |  |  |
| Urine RBC counts | 1.002(0.997-1.005) | 0.215 |  |  |
| Viral load (log_10_ copies/mL) | 4.631(3.033-7.070) | <0.001 | 3.011(1.618-5.605) | 0.001 |
| OBT positivity, n (%) | 1.863(0.935-3.710) | 0.077 |  |  |
