# Supplementary material for: Prevalence and clinical characteristics of increased pancreatic enzymes in patients with severe fever with thrombocytopenia syndrome
Source: PLoS Negl Trop Dis. 2023 Nov 9;17(11):e0011758. doi: 10.1371/journal.pntd.0011758 (PMC10662747; doi:10.1371/journal.pntd.0011758)
Supplement: S1 Text — (DOCX) [file pntd.0011758.s005.docx]

**Glossary**

| AP | acute pancreatitis |
| --- | --- |
| ALT | alanine aminotransferase |
| AST | aspartate aminotransferase |
| ALP | alkaline phosphatase |
| APTT | activated partial thromboplastin time |
| BNP | brain natriuretic peptide |
| BUN | blood urea nitrogen |
| CK | creatinine kinase |
| CK-MB | creatinine kinase myocardial b fraction |
| CRP | C-reactive protein |
| ESR | erythrocyte sedimentation rate |
| GGT | gamma glutamyl transpeptidase |
| INR | international normalized ratio |
| IL-6 | interleukin-6 |
| LDH | lactate dehydrogenase |
| OBT | occult blood test |
| PT | prothrombin time |
| PTA | prothrombin activity |
| SFTS | severe fever with thrombocytopenia syndrome |
| TBIL | total bilirubin |
| TT | thrombin time |
| WBC | white blood cell |
