# Supplementary material for: Prevalence and clinical characteristics of increased pancreatic enzymes in patients with severe fever with thrombocytopenia syndrome
Source: PLoS Negl Trop Dis. 2023 Nov 9;17(11):e0011758. doi: 10.1371/journal.pntd.0011758 (PMC10662747; doi:10.1371/journal.pntd.0011758)
Supplement: Attachment — Submitted filename: Response to reviewers.docx [file pntd.0011758.s006.docx]

Reviewer #1: General comments

The authors studied the prevalence of acute pancreatitis (AP) and the outcomes among 284 patients diagnosed as having severe fever with thrombocytopenia syndrome (SFTS). The authors divided the 284 patients into 3 categories (groups) based on the level of amylase and/or lipase, high level (more than 3 times of upper limit of normal (ULN)), middle level (more than the ULN to less than the high level), and normal within the normal level. It is of importance to study the association between clinical course and outcomes of patients with SFTS and the level of pancreatic enzymes. The authors reported that the higher level of pancreatic enzymes, amylase and lipase, in patients with SFTS was closely associated with disease severity and outcomes. Even in the SFTS patients with high level of pancreatic enzymes, the patients with AP had lower survival rate than those without AP.

This reviewer considers that the criteria for diagnosing the patients as having AP is very critical and important for this study. It is written that patients were diagnosed as having AP when the patients had at least 2 of the following 3 criteria, 1) abdominal pain, 2) serum amylase and/or lipase levels (>= 3 times of ULN), and 3) characteristic findings of AP on imaging examinations. However, the AP-diagnostic status for the 284 patients were unclear. This reviewer wishes to know what kind of imaging tests were used, CT, MRI, or others. It should be clarified. This reviewer also wishes to know how many patients were tested for imaging examination. This reviewer considers that it is necessary how many patients were diagnosed as having AP with 1) and 2) criteria, the1) and 3), 2) and 3), and 1), 2) and 3). This reviewer considers that there is no significant difference in the discrimination between the criteria with the combination of 1) and 2) and the combination of 1) and elevated amylase and/or lipase between 1 × ULN and 3 × ULN.

Response: We greatly appreciate your recommendations to elaborate the AP-diagnostic status for the 284 patients. In our study, CT was used for imaging tests. A total of 136 patients were tested for imaging examination, including 16 patients with abdominal pain and amylase and/or lipase <3×ULN, 31patients with abdominal pain and amylase and/or lipase >3×ULN, 89 Patients without abdominal pain but with amylase and/or lipase >3×ULN. These details highlighted in yellow have been updated in the **Diagnostic criteria** sections (page 5, line 100-104). According to your kind comments, the clinical data of 36 patients diagnosed with AP were reviewed. Their diagnosis patterns of AP were as follows: five patients in line with 1+2 criteria, three patients in line with 1+3 criteria, 28 patients in line with 1+2+3 criteria. These details highlighted in yellow have been added in the **Results** sections (page 10, line 180-182). In the present study, a total of 36 patients were diagnosed with AP, and their amylase and/or lipase were all higher than 3 times of ULN. Thus, there was no patients diagnosed with AP that met the criteria with the combination of 1) and elevated amylase and/or lipase between 1 × ULN and 3 × ULN.

Specific comments

1) If the line numbers were written, it would be helpful for the reviews to make comments.

Response: Thanks for your significant suggestions, the line numbers have been written in the manuscript.

2) Page 2, abstract: “then their clinical data were compared” should be “and then their clinical data were compared”.

Response: According to your suggestions, abstract: “then their clinical data were compared” has been corrected to be “and then their clinical data were compared”.

3) Page 2, abstract: The sentence, “Higher viral load was also detected in the HPE group”, does not make sense. “than what”?

Response: According to your comments, the sentence “Higher viral load was also detected in the HPE group” has been corrected to be “as well as higher viral load”. We aimed to show that higher SFTS viral load was also detected in the HPE group than that in the normal and EPE groups.

4) Page 3: The description, “an emerging zoonosis infected by SFTS virus (SFTSV)”, does not make sense. A disease (zoonosis) can not be infected with a virus. Human (patient) is infected with a virus.

Response: Thanks for your kind suggestions, the description, “an emerging zoonosis infected by SFTS virus (SFTSV)” has been corrected to be “an emerging zoonosis caused by SFTS virus (SFTSV)”.

5) Page 4-5, Diagnostic criteria: Please refer the comments in the General comments.

Response: According to your comments in the general comments, the details were updated in in the **Diagnostic criteria** sections (page 5, line 100-104).

6) Page 7: The description, “frequency of diabetes ---- ----encephalopathy” needs correction. Symptoms and disease pathophysiology are in the same line. How was the pathophysiology of encephalopathy confirmed in patients with SFTS? It is recommended that “frequency of diabetes” would be replaced by “frequency of having diabetes melitus”.

Response: According to your significant suggestions, the description, “encephalopathy” has been replaced by “consciousness disorder”, including drowsiness, confusion, lethargy and coma. The description, “frequency of diabetes” has been replaced by “frequency of having diabetes mellitus”.

7) Page 7: The description “38℃” should be “38 ℃”. Please insert a space. This correction is needed in the entire text including tables.

Response: Thank you for your kind comments, the description “38℃” has been insert a space, and this correction has been shown in the entire text including tables.

8) Page 9: “difference of other clinical features” should be “difference in other clinical features”?

Response: Thank you for your kind comments, the description “difference of other clinical features” has been corrected to be “difference in other clinical features”.

9) In general, there are several multiple descriptions in the entire text. For instance, the description that the authors reported the association between the disease severity and serum amylase/lipase levels were elucidated for the first time to the authors’ knowledge appeared several times.

Response: According to your important suggestions, once a description was shown in the text, it was not been appeared again.

10) Discussion section, page 15, last sentence: Is it acceptable to use the term “perfect” for this discussion? This reviewer does not consider so.

Response: We greatly agree with your suggestions, the description “and their diagnostic performance was perfect” had been deleted in the **Discussion** section.

Reviewer #2: Although this study is performed well, I have major issues with the lack of confounding as described above. The paper in its current form is not enough to inform clinical practice. The paper will be significantly improved if the analysis is redone without acute pancreatitis patients. This way, one can truly assess the association betweek elevated pancreatic enzymes and clinical outcomes in SFTS.

Response: Thank you for your kind comments. When we conducted a retrospective study on the clinical characteristics and prognosis of patients with SFTS, we found that elevated pancreatic enzymes were very common in SFTS patients. Though SFTS patients didn’t have abdominal pain or imaging tests results, they would be diagnosed with “AP” and treated with inhibition of gastric acid secretion, somatostatin, octreotide and intravenous antibiotics if their pancreatic enzymes were higher than 3×ULN. However, they didn’t have AP in fact, and they would not be benefited from the clinical practice. Thus, it is critical for clinicians to discriminate AP from SFTS patients with elevated pancreatic enzymes. However, to our knowledge, there is no study to report the incidence of increased pancreatic enzymes and its association with AP in patients with SFTS.

To our knowledge, at present, there is only a study reported the clinical features and outcomes of AP in SFTS (Tian B, et.al, Pancreatology, 2020). However, in the study, only a total of 14 patients were diagnosed with AP, and the relationship between elevated pancreatic enzymes and AP was not described. Most importantly, owing to small number of AP cases, it didn’t describe minutely the impact of AP on outcomes of patients with SFTS and show that AP is an independent risk factor for prognosis of patients with SFTS.

Our study originally demonstrated that increased pancreatic enzymes was very common in patients with SFTS, but it was not always appeared in AP. Though patients with elevated pancreatic enzymes showed high in-hospital mortality, it is AP to account for the majority of their death. The aim of the study was to emphasize that elevated pancreatic enzymes may not always represent a true AP, clinicians should confirm the diagnosis of AP by means of imaging examinations in patients with SFTS.

According to your important suggestions, we explored the clinical characteristics of elevated pancreatic enzymes without acute pancreatitis and its impact on prognosis in patients with SFTS. These results had been updated in the manuscript.

A total of 284 patients were assigned into the non-AP and AP groups, and then patients in the non-AP group were divided into the normal (<ULN), elevated pancreatic enzymes (EPE) (1-3×ULN) and high pancreatic enzymes (HPE) (>3×ULN) groups according to the serum amylase and/or lipase levels. The demographics, comorbid conditions, clinical manifestations, laboratory tests results and cumulative survival rates of SFTS patients in the normal, EPE and HPE groups were compared.

The cumulative survival rate of patients in the normal group was significantly lower than that of patients in the HPE group (*P*=0.034). However, there was no significant difference in the cumulative survival rates between the normal and EPE groups (*P*=0.198), as well as the same result was observed between the EPE and HPE groups (*P*=0.169).

The demographics, comorbid conditions, clinical manifestations, laboratory tests results and cumulative survival rates of SFTS patients in the HPE and AP groups were also compared. Patients in the AP group also had higher serum levels of laboratory variables reflecting liver, heart, coagulation dysfunction and viral load than patients in the HPE group. The cumulative survival rate of patients in the AP group was significantly lower than that of patients in the HPE group.

In summary, the increased pancreatic enzymes are very common in patients with SFTS, but they are not always associated with AP. Imaging examinations could be necessary for clinicians to confirm the diagnosis of AP when SFTS patient with elevated pancreatic enzymes. Though AP may account for the majority of mortality of patients with elevated pancreatic enzymes, patients with pancreatic enzymes >3×ULN except for AP also have a high in-hospital mortality rate.
